# An Insight into Anticancer Effect of Propolis and Its Constituents: A Review of Molecular Mechanisms

**DOI:** 10.1155/2022/5901191

**Published:** 2022-06-17

**Authors:** Perumal Elumalai, Natarajan Muninathan, Sadhasivan T. Megalatha, Arumugam Suresh, Kalimuthu Senthil Kumar, Nathan Jhansi, Kuppuswamy Kalaivani, Gunasekaran Krishnamoorthy

**Affiliations:** ^1^Center for Transdisciplinary Research, Department of Pharmacology, Saveetha Dental College, Saveetha Institute of Medical and Technical Sciences, Chennai, Tamil Nadu, India; ^2^Central Research Laboratory, Department of Biochemistry, Meenakshi Medical College Hospital and Research Institute, MAHER University, Enathur, Chennai, Tamil Nadu 631552, India; ^3^Central Research Laboratory, Swamy Vivekanandha Medical College Hospital and Research Institute, Elayampalayam, Tiruchengode, Namakkal, Tamil Nadu 637205, India; ^4^Zebrafish Developmental Biology Laboratory, AUKBC Research Centre, Anna University, Chromepet, Chennai 600044, India; ^5^Research Center for Cellular Genomics and Cancer Research, Sree Balaji Medical College and Hospital, Chromepet, Chennai 44, India; ^6^Department of Medical Biochemistry, College of Health Sciences, Dambi Dollo University, Dembi Dollo, Ethiopia

## Abstract

Propolis is a natural compound collected by honeybees from different parts of plants. Honeybees produce a sticky component besides honey by mixing the tree resin and other botanical sources with saliva called propolis or bee glue. Propolis was traditionally used as a wound healing substance, cosmetic, medicine, and many other conditions. Till now, there is no definite curable treatment for most cancers and chemotherapeutic drugs and drugs used for targeted therapies have serious side effects. According to a recent research, natural products are becoming increasingly essential in cancer prevention. Natural products are a great source of potential therapeutic agents, especially in the treatment of cancer. Previous studies have reported that the presence of caffeic acid phenethyl ester (CAPE), artepillin C, and chrysin is responsible for the anticancer potential of propolis. Most of the previous studies suggested that propolis and its active compounds inhibit cancer progression by targeting multiple signaling pathways including phosphoinositide 3-kinases (PI3K)/Akt and mitogen-activated protein kinase (MAPK) signaling molecules, and induce cell cycle arrest. Induction of apoptosis by propolis is mediated through extrinsic and intrinsic apoptotic pathways. The aim of this review is to highlight and summarize the molecular targets and anticancer potential of propolis and its active compounds on cell survival, proliferation, metastasis, and apoptosis in cancer cells.

## 1. Introduction

Cancer is the world's second major cause of death and a serious global health issue. In 2020, an estimated 19.3 million new cancer cases were diagnosed worldwide, with over 10 million cancer deaths. An expected 2.3 million new cases of female breast cancer have surpassed lung cancer as the most commonly diagnosed malignancy, followed by lung, colorectal, prostate, and stomach cancers [1]. Cancer is a multifaceted disorder relating to abnormal cell growth, and cancer cells form aggressive unwanted masses of tissue called tumors, which are capable of spreading to other organs through the bloodstream or lymph system to form metastatic cancer [2]. Cancer cells dedifferentiate and are able to grow aggressively and escape from growth regulatory signals such as cell cycle control and apoptosis [3]. Currently, there are different methods for cancer treatment available based on cancer type and grade, such as radiation and/or chemotherapy and other options like including immunotherapy, targeted therapy, and hormone therapy employed in cancer patients [4–7]. The chemotherapeutic approach is used for the treatment of benign and malignant cancers; however, it has several limitations like side effects and off-target effects [8, 9].

Cancer tissue contains heterogeneous cell populations, and these cells activate multidrug resistance, thereby triggering tumor relapse. The available common cancer treatments have no complete cure and cause various side effects. Often chemotherapeutic drugs have poor bioavailability and a narrow therapeutic index. Therefore, there is an immediate requirement for the development of natural compound-based anticancer drugs with target-specific, cost-effective, and minimal side effects for cancer treatment. Previously, numerous research studies have been conducted to identify anticancer drugs from natural products [10, 11]. Several naturally obtained drugs like paclitaxel, vinblastine, vincristine, and several others are being clinically used as anticancer medicines. Therefore, natural products are developed as an essential research field, to identify the potential novel anticancer agent and also to understand the mechanism of action on cancer cells. Hence, this review summarizes the molecular targets and anticancer properties of propolis and its active compounds against various cancers.

## 2. Propolis

Propolis is a natural resinous substance produced by honeybees from plant-based natural source materials like tree buds, sap flowers, etc., by mixing with saliva secretions. It is used for the protection from infections, to seal gaps, openings in the hive, and toughen combs, and also, propolis is used to maintain the hive's internal temperature, leveling the beehive surface to protect from abnormal weather and predators [12]. Honeybees are flying insects, closely related to wasps and ants. The honeybee products like honey, beeswax, bee venom, and bee pollen have been used as traditional medicine and also exhibited antimicrobial, antioxidant, anti-inflammatory, and anticancer properties in several preclinical studies in cell line and animal experiments [13–17]. The word “Propolis” comes from Greek that means defense for “pro” and city or community for “polis,” or the beehive in other words. Propolis has been used by humans for many years as a powerful herbal and dietary supplement. The nature of propolis is like plastic material that protected the human body after death from bacteria, fungi, and viruses, and the Egyptians used it as preservative material to embalming the human body [18]. The presence of phytochemicals (polyphenols, flavonoid, phenolic acids, phenolic aldehydes, and ketones) in propolis is responsible for its biological properties, including antioxidant [19], anti-inflammatory [20], and anticancer potential [21–23]. The types of propolis are classified based on their origin, color, and texture. Generally, Asians and Europeans use propolis largely in the management of many diseases [24, 25].

### 2.1. Chemical Composition and Biological Properties of Propolis

The propolis is multifaceted, and the chemical composition differs according to bee species, geographical origin, and plants that stimulate the bee's biological activities [26, 27]. Different type of propolis has a variety of biological properties including antioxidant and anticancer effects based on their origin and chemical composition [28, 29]. The presence of polyphenols and flavonoids determines the therapeutic properties of propolis, and interestingly, there are 38 different types of flavonoids present in the propolis. The propolis contains 24 to 26% of fatty acids, 18 to 20% of flavonoids, 0.5 to 2% of microelements, 15 to 18% of sugars, 5 to 10% of aromatic acids, 2–6% of esters, 2 to 3.3% of alcohol and terpenes, and 2 to 4% of vitamins [30, 31]. Resins and balms are major components (50–60%) in raw propolis. Also, waxes (30–40%), essential oils (5–10%), pollen (5%), and vitamins (5%) are present in propolis [32, 33]. It has been reported that 300 different types of polyphenols compounds [34], terpenoids [35], amino acids, and others are found in propolis [32]. The HPLC-MS (high-performance liquid chromatography-mass spectrometry) and ultra-PLC-MS analyses have identified the predominant bioactive components in propolis such as chrysin, CAPE, artepillin C, nemorosone, galangin, cardanol, cardol, quercetin, kaempferol, and p-coumaric acid in Brazilian green propolis [36].

Propolis polyphenolic compounds are having anticarcinogenic activity, and in addition to this, well-known predominant bioactive components of propolis like CAPE, artepillin C, and chrysin can inhibit cancer cell growth in various malignancies [27, 37–39]. Artepillin C, another form of propolis, is reported to induce cytotoxicity through activating apoptosis and necrosis and inhibits mitosis in melanoma and carcinomas cells [1]. Chrysin has an anticancer property by inducing extrinsic and intrinsic apoptosis through activating caspases, and inhibiting antiapoptotic proteins in cancer cell lines [40]. The propolis composition differs based on the plant origin. For example, New Zealand propolis contains CAPE, Brazilian propolis contains artepillin C, and propolin G in Taiwan propolis [41]. Okinawa propolis (OP) originated from *Macaranga tanarius* comprises prenylated flavonoids [42]. Several recent studies also reported that the anticancer properties of propolis and its active principle compounds such as CAPE, artepillin C, and chrysin have shown anticancer potential by apoptosis induction and inhibition of cell growth in different cancer cells [43–46].

It has been established that prenylated flavonoids from OP could strongly suppress the albumin denaturation, thereby preventing the chronic inflammation-mediated cellular protein denaturation. Furthermore, OP treatment decreases the COX-2 activity and nitrite accumulation in lipopolysaccharide-induced inflammatory model, RAW264.7 cells [47]. Earlier reports elucidate that the antidiabetic, anti-inflammatory, and anti-Alzheimer's drug properties of OP are mediated through inhibition of PAK1 signaling. Overall, all of the earlier findings suggested that components of OP could be a harmless and novel drug for Alzheimer's, diabetes, and inflammation treatment [47]. Elnakady et al. [48] studied the importance of Saudi Arabia propolis in cancer cells, in which they have demonstrated that the propolis-treated cells showed inhibition of cell proliferation and induction of apoptosis. Furthermore, their gas chromatography-mass spectrometry (GC-MS) examination confirmed the occurrence of an active fraction, i.e., Saudi propolis L-acetate, and it was isolated from the region of Al Bahah. The occurrence of triterpenoids such as lupeol, ferruginol, *β*-amaryl acetate, *β*-amyrins, and lupeol acetate, sugiol, taraxasterol, and totarol is implicated in their beneficial effect on Saudi propolis. These fractions had the potential to inhibit the tubulin activity through cell cycle arrest at the G2M phase and to induce apoptosis in cancer cell lines.

Although propolis is a good source of minerals, amino acids, vitamins A, E, and B complex, and the biochemical compounds like bioflavonoids, phenols, aromatic compounds, and many other molecules, which possess hepatoprotective activity, anti-inflammatory, antiproliferative, antifungal, antibacterial, and antimicrobial qualities, the exact constituent of propolis is still unknown; hence, further research is required to find out the new compounds [31, 49–51]. The Brazilian red propolis is seen in red color due to pigmentation of Retusapurpurin B and Retusapurpurin A flavanols, and their chemical composition and pharmacological activities were thoroughly studied in a recent study [52]. By considering the above properties, propolis is utilized in manufacturing following cosmetics like soap, perfume, and sunscreen [31, 53]. It is also used in mouthwash for oral care for patients suffering from chemotherapy to improve oral hygiene [54].

**Figure 1:**
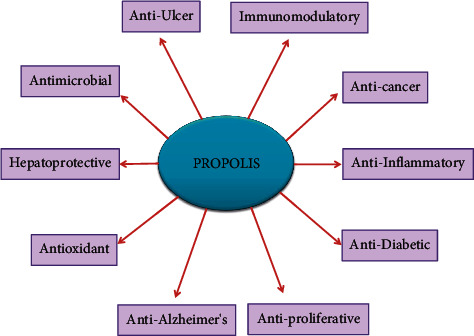
Overview of the biological properties of propolis and its bioactive components.

Several studies have demonstrated that the antioxidant activity of flavonoids was achieved through the potential to decrease the formation of free radical and scavenging capacity [30, 55]. The antioxidant potential of propolis is well studied, and its powder is commercially available as a supplement that increases antioxidant enzymes like glutathione peroxidase, superoxide dismutase, and catalase [56, 57]. Recently, propolis was shown to have anti-inflammatory and immunomodulatory activity and inhibits the PAK1. In addition, propolis inhibits the interaction of ACE2 and SARS-CoV-2 virus. In silico approach reported that active components of propolis, such as CAPE, quercetin, rutin, kaempferol, and myricetin, possess a high affinity with ACE2. Furthermore, propolis does not affect the key enzymes and liver function; hence, it can be a better drug without any side effects [58]. Further investigations and studies are urgently needed to understand the exact mechanisms of propolis in the future.  Figure 1 shows the biological properties of propolis and its bioactive components.

### 2.2. Bioavailability of Propolis

Poor gastrointestinal absorption and bioavailability hinder the effectiveness of numerous natural and synthetic anticancer drugs during the drug development process. The failure of drugs in clinical trials is frequently due to poor absorption and oral bioavailability of drugs [59].

It is crucial to understand which propolis components are bioavailable in the human body. Because most of the activity is given by free forms, and conjugation makes it harder to demonstrate activity, bioavailability is directly connected with sensitivity to conjugation when the components are integrated through the intestines [60]. To evaluate the in vivo antioxidant capabilities of propolis and its effect on human health, the researchers first investigated its bioavailability. These findings indicated that galangin may be adsorbed and promptly glucuronidated after oral treatment, and a brief bioavailability in mice for galangin, chrysin, and its derivatives [60].

Pharmacokinetic properties of compounds present in Okinawa propolis (OP) were analyzed by molinspiration online toolkit to consider them as potent drug candidates based on its Lipinski's 5^th^ rule [61]. All the OP compounds have better absorption and bioavailability [47]. Several studies proved that the pharmacokinetics and toxicological properties of propolis approved them as potential drug sources [62]. According to Kimoto et al., [63, 64] an antioxidative dosage of artepillin C prevented renal and pulmonary malignancies in male mice developed by ferric nitrilotriacetate. As a result, artepillin C, which is difficult to conjugate and has a strong affinity for cell membranes, is a potential bioavailable factor for use in degenerative disease chemoprevention.

Polyphenols are known to have limited bioavailability as a result of their molecular weight, glycosylation level, metabolic conversion, and contact with the gut bacteria, which limits their use in vivo. The goal of the most recent study was to synthesize poly-(lactide-co-glycolide) acid (PLGA) nanoparticles for the encapsulation of Sonoran propolis (SP) as a matrix to increase the solubility of their polyphenolic compounds and improve delivery, and assess its antiproliferative activity on cancer cells. The SP might be possibly employed in antitumoral in vivo experiments after being encapsulated into PLGA nanoparticles, since the resulting delivery system displays suitable encapsulation features and antiproliferative activity for usage in the field of nanomedicine [65]. The activity of CYP450 enzymes is crucial for therapeutic effectiveness since it has a major impact on the drug's concentration in circulation and its metabolites. If a drug's efficacy is mostly determined by its original form (rather than its metabolites), inhibiting its corresponding CYP450 enzyme activity results in lower biotransformation activity, resulting in stronger drug effects or even overdose-induced toxicities. Induction of CYP450 enzyme activity, on the other hand, may result in a diminished drug effect and effectiveness [66]. Propolis is a combination of bioactive chemicals that are largely metabolized by the CYP450 family, and its impact on CYP450 is becoming more studied. As a result, it raises concerns about possible side effects from mixing propolis with other drugs owing to changes in CYP450 enzyme activity.

The activity levels of CYP1A2, CYP2C9, CYP2C19, CYP2D6, and CYP3A4 in human hepatocytes were tested by Sasaki et al. [67]. A propolis-containing product, for instance, was shown to inhibit all five CYP450s by more than 50%. Naramoto et al. [68] examined studied blood concentrations of essential bioactive compounds in propolis to see whether they were high enough to elicit a clinical change in CYPs in the body. As the principal bioactive components, artepillin C, kaempferide, dihydrokaempferide, isosakuranetin, and kaempferol were shown to contribute to the CYP450 inhibitory activity of a standardized propolis extract (EEP-B55). The bioavailability of these main bioactive chemicals was then examined. Because of their low concentration in blood and hepatocytes, their ability to inhibit CYPs in the body was considered insignificant. Cusinato et al. [69] partially justified this hypothesis, revealing that the effects of propolis extract on CYP1A2, CYP2C9, CYP2C19, CYP2D6, and CYP3A in healthy adult volunteers were not significant. Though multiple preclinical investigations showed that coincubation of propolis extract modified the CYP450 enzymes, the human trial revealed that the effect of propolis on CYP450 enzymes was modest due to the limited bioavailability of contributing compounds found in the propolis. Propolis phytoconstituents differ depending on the source. To guarantee consistent quality and effectiveness control of the propolis product, standardization of the chemical composition of propolis extract is required.

## 3. Molecular Mechanism of Anticancer Effect of Propolis and Its Bioactive Compounds

### 3.1. Effect of Propolis and Its Bioactive Compounds on Cancer Progression

Previous preclinical studies have reported that major compounds in the propolis such as flavonoids and polyphenols are responsible for its chemopreventive and anticancer effects [70, 71]. The anticarcinogenic activity of the flavonoid compounds in propolis can repress angiogenesis and induce apoptosis, thereby it act against tumor growth [72]. The chemopreventive effect of propolis in several cancer types is mediated by inhibiting tumor growth and induction of apoptosis [55] further, and in another way, it scavenges the reactive oxygen species and stimulates the activity of antioxidant enzymes [73]. Due to the side effects and drug resistance of anticancer drugs, the recent research on cancer is focusing on natural sources like propolis. The Algerian propolis extract-treated cells showed reduced cell proliferation and inhibited the cell adhesion by altering the fibrinogen in lung cancer [74].

The Wnt signaling is a primordial and evolutionarily conserved pathway that controls cell differentiation, proliferation, and migration [39]. The APC and Axin are Wnt pathway suppressors, and another downstream molecule *β*-catenin is involved in cancer progression. *β*-catenin gene mutation is found in many cancers. The CAPE administration in HCT16 and human colon cancer cell lines suppressed the Wnt signaling pathway [29]. Chrysin increases p21 protein expression by stimulating the p38 mitogen-activated protein kinase activity that led to the inhibition of the cell cycle and suppresses the cell proliferation in of C6 glioma [28]. Chrysin treatment in U937 cells (histiocytic lymphoma cells) showed induction of apoptosis by suppressing the PI3K/Akt signaling and inactivation of nuclear factor kappa B (NF-*κ*B)/inhibitor of apoptosis (IAP), which led to the activation of caspase-3, which triggered apoptosis [75]. Another latest research reported that Turkey originated propolis extract can have antiproliferative, apoptotic, and cell cycle arrest potential against cancer cell lines. The presence of 3‐O‐methylquercetin, CAPE, galangin, chrysin, caffeic acid, and pinocembrin was implicated in the anticancer potential [76]. The CAPE exhibits cytotoxic and antiproliferative effects against breast cancer cell lines by increasing the endothelial nitric oxide synthases (e-NOS) and inducible nitric oxide synthase (i-NOS) levels [77]. The CAPE treatment showed the suppressed extracellular signal-regulated kinase (ERK) and Akt phosphorylation and decreased cyclin D expression in 3T3-L1 fibroblast cells [12]. Ethanol extract of propolis treatment significantly decreased the proliferation at 70 and 22 *μ*g/ml in DU145 and PC3 cells. Also, it induces the cell cycle arrest by altering the cell cycle regulatory proteins including cyclins and cyclin-dependent kinases [78]. The effect of CAPE (75 *μ*M/ml) on cell cycle-related gene expressions (CCND2, RB1, ATM, CDC34, and CDK5RAP1) was evaluated by real-time PCR in breast cancer cells. The CAPE significantly increased the expression of cell cycle regulatory genes when compared to control cells, thereby it may induce the cell cycle arrest in breast cancer [40].

**Figure 2:**
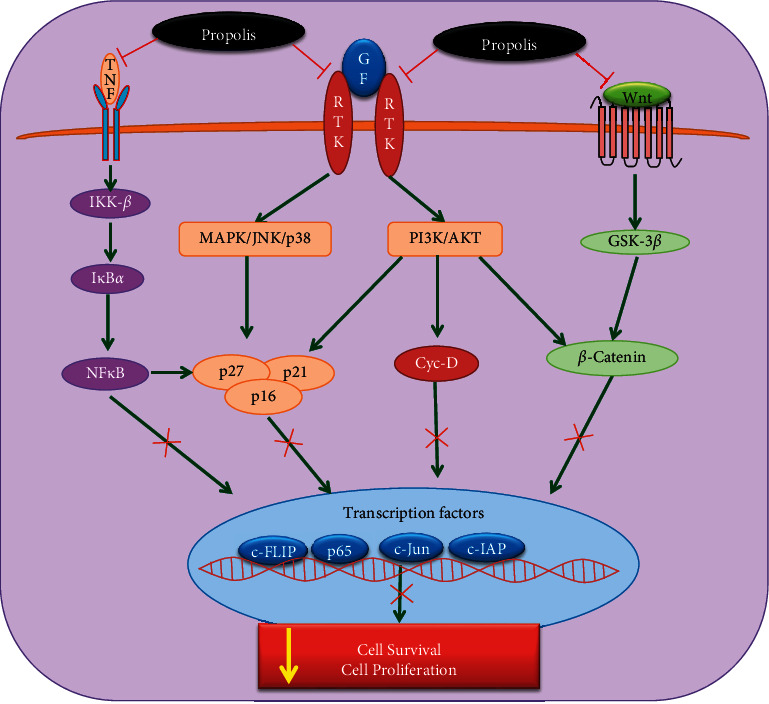
The molecular targets of propolis and its bioactive compounds in inhibition of cell proliferation and cell survival and induces the cell cycle arrest in cancer cells. The green arrow points out the upregulation of signaling molecules, while the red arrow indicates the downregulation of molecular targets. GF: growth factor, RTK: receptor tyrosine kinase, MAPK: mitogen-activated protein kinases, p38: p38 mitogen-activated protein kinases, JNK: c-Jun N-terminal kinase, PI3K: phosphoinositide 3-kinase, AKT: protein kinase B PAK1: p21-activated kinase-1, p16, p21, p27, and p16: cyclin-dependent kinase inhibitors, NFkB: nuclear factor-*κ*B, IKK: inhibitor of NF-*κ*B kinase, CDK: cyclin-dependent kinase, c-FLIP: cellular FLICE-inhibitory protein, c-IAP: cellular inhibitor of apoptosis protein 1.

The CAPE significantly repressed the activation of signal transducer and activator of transcription-3 (STAT-3) in HEp-2 cells. Further investigation using AG490, a selective STAT-3 inhibitor, demonstrated that CAPE not only suppressed STAT-3 but also suppressed the expression of PLK-1 in HEp-2 cells, indicating that STAT-3 was likely involved in the regulation of PLK-1 in HEp-2 cells. Furthermore, CAPE exposure caused a disruption of the cell cycle in the S phase in HEp-2 cells by inhibiting the STAT-3/PLK-1 pathway [45]. A recent study demonstrated that Chinese propolis (25, 50 & 100 *μ*g/ml) treatment inhibits cell proliferation by targeting glycolysis enzymes and proinflammatory cytokines including TNF-*α*, IL-6, and NLRP3 in the breast cancer cells [79]. Reports from a recent study reveal that CAPE blocks the expression of the MALT1 gene to decrease the cell proliferation, invasion, and tumor growth of prostate carcinoma cells via the p53 and NF-*κ*B signaling pathways, and they further verify that CAPE is an effective antitumor agent for human androgen-dependent and androgen-independent prostate carcinoma cells by inhibiting MALT1 expression in vitro and in vivo [80]. Overall, all of these studies suggested that propolis and its constituents inhibit cell proliferation, survival, and cell cycle by altering various signaling pathways (PI3K/Akt, MAPK, TNF-*α*/NF-*κ*B, Wnt/*β*-catenin, and STAT-3/PLK-1) in several cancers (Figure 2).

The translation of preclinical data to a human application is the most critical aspect of any therapeutic's development. Propolis has been carefully studied for decades and has been utilized as a folk medicine for thousands of years. However, there is currently a lack of human clinical data, particularly in the field of cancer treatment. Propolis' possible preventive effect in breast cancer patients receiving chemotherapy and radiation was examined by Ebeid et al. [81]. This clinical trial comprised a total of 135 individuals. Propolis capsules (400 mg, 3 times daily) is consumed for 10 days before radiotherapy, 10 days during radiation treatment, and 10 days after irradiation. Propolis was reported to reduce the harmful effects of radiation in breast cancer patients, such as a rise in Comet tail parameters in peripheral blood mononuclear cells and serum malondialdehyde (MDA). Propolis also reduced radiotherapy-induced reductions in total antioxidant capacity, hemoglobin (Hb) concentration, white blood cells (WBCs), and platelet counts. Patients who used propolis supplements had a considerably longer median disease-free lifetime. This study found no adverse effects associated with propolis consumption [81].

Another pilot randomized clinical experiment was conducted to assess the safety, toxicities, and compliance with propolis in breast cancer patients receiving doxorubicin and cyclophosphamide, and test the preliminary clinical efficacy of propolis in the prevention of chemo-induced oral mucositis (OM) and prospectively assessing the incidence of OM. Sixty patients were randomly assigned to receive either a dry propolis extract containing 8%–12% galangin plus mouth rinsing with sodium bicarbonate or mouth rinsing with sodium bicarbonate. The incidence of OM was also studied throughout a 6-month period. Propolis with bicarbonate was shown to be safe, well tolerated, and effective in preventing OM in breast cancer patients [82].

Patients' nutritional health and quality of life suffer as a result of chemotherapy-related side effects. Propolis has been proposed as a coadjuvant nutritional supplement in cancer treatment due to its functional characteristics and biological activities, such as antitumoral activity, DNA protection, free radical scavenging, and immune stimulation; however, clinical trials to support these effects in cancer patients are required. The findings of a recent randomized, double-blind, and placebo-controlled clinical trial compared propolis to a placebo on nutritional status and quality of life in breast cancer patients after chemotherapy. Propolis has been indicated as a viable and safe therapeutic option for breast cancer patients receiving chemotherapy who want to improve their nutritional condition and quality of life [83]. Propolis, as an adjuvant therapy, may help to alleviate the symptoms of breast cancer. However, further clinical studies are urgently needed, particularly with a larger number of participants/subjects/patients.

### 3.2. Apoptosis-Inducing Potential of Propolis and Its Bioactive Compounds in Cancer Cells

Induction of apoptosis is a major mechanism proposed for the therapeutic effect of cancer drugs. Apoptosis is a programmed cell death regulated by two different pathways: 1. extrinsic pathway: activated by external factors like TNF-*α* (tumor necrosis factor-*α*), FasL (Fas ligand), and TNF-related apoptosis-inducing ligand (TRAIL); 2. intrinsic or mitochondrial-mediated pathway: this pathway is induced by ROS, which disrupts the mitochondrial membrane potential that leads to activation of proapoptotic proteins (p53, Bax/Bad, and cytochrome c) [84]. The anticancer potential of water-soluble derivatives of propolis (WSDP) from Brazil and Croatia was reported on MCF-7 (breast cancer), HeLa (cervical cancer), and V19 (lung fibroblast hamster) cells. Brazilian and Croatian propolis (50 *μ*g/ml)-treated cells showed a significantly increased apoptotic percentage of MCF-7 and HeLa cells as compared to the control and V19 normal fibroblasts. The results from the above study demonstrated the effect of propolis in different cancer cells and normal fibroblast cells [85]. The antiproliferative/antitumor effect of propolis is studied *in vitro* and *in vivo*, where it showed its capability to suppress DNA synthesis in cancer cells and the capacity to promote apoptosis. The CAPE has the ability to promote apoptosis in breast cancer cell lines (MCF-7 and MDA-MB-231) [86]. Propolis extracts have shown apoptosis-promoting potential against diverse cancer cell lines such as HeLa [87], prostate adenocarcinoma [88], basophilic leukemia [89], and human breast [90], to study the activation of caspase and inhibition of mast cell granulation [91]. Ethanolic propolis extract (250 or 500 *μ*g/ml) treatment induces apoptosis in C6 glioma cells by increasing the mRNA expression of caspase-3, caspase-8, and caspase-9. Also, it increases the total antioxidant levels and glutathione levels in glioma cells [92].

Propolis and CAPE inhibit the growth and migration of breast cancer cell lines. Furthermore, propolis modulates the inflammatory microenvironment and induces apoptosis by inhibiting TLR4 singling [93]. In another study, Turkish ethanolic propolis extract induces apoptosis by upregulating proapoptotic protein levels and alters the oncogenic and tumor suppressor gene expression in breast cancer cell lines [94]. A recent report suggested that red propolis along with L-lysine reduces tumor angiogenesis and tumor growth in hamster cheek pouch walker 256 cancer cell-inoculated cancer model [95]. Cuban propolis extracts and active compound nemorosone inhibit cell proliferation and induce apoptosis in doxorubicin-resistant colon cancer cells. These results showed that nemorosone alone was able to suppress cell proliferation. The combination of propolis extracts with nemorosone induces cell cycle arrest and decreases cell growth and also promotes apoptosis by ROS production and dissipation of mitochondrial membrane potential [96]. The chrysin acts as a possible replacement for the 5-FU and oxaliplatin combination to attain treatment strategy through autophagy for colorectal cancer therapy in the future [18]. The CAPE (1–30 *μ*M) suppressed the growth of human multiple myeloma cells while leaving normal peripheral blood B cells unaffected. The CAPE induction of apoptosis in multiple myeloma cells was validated by flow cytometry, with up to 50% apoptotic cells induced by 50 *μ*M CAPE within 24 h by activating the apoptosis executioner enzyme caspase-3 and corresponding cleavage of PARP. The oxidative stress generated by CAPE cytotoxicity in multiple myeloma cells was assessed using ROS and antioxidant levels. All of this evidence suggests that CAPE induces apoptosis in human multiple myeloma cells via oxidative stress [59].

**Figure 3:**
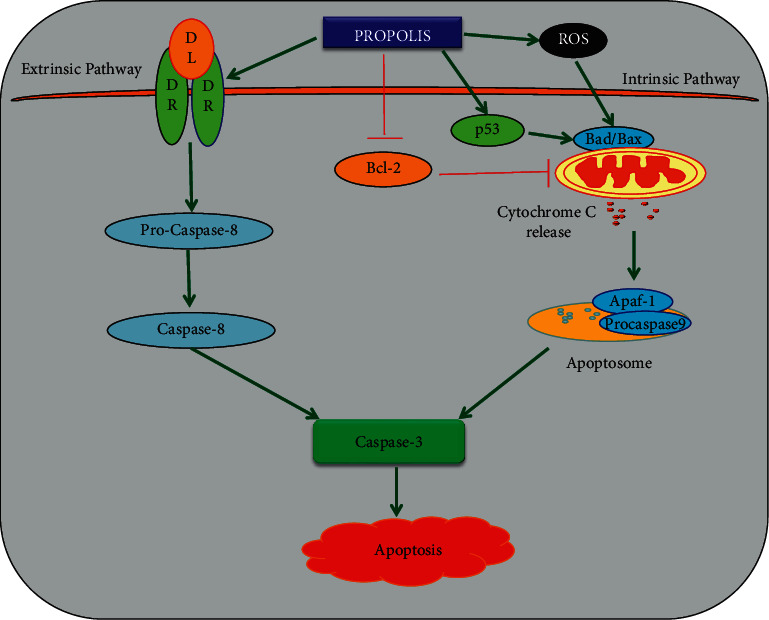
Propolis and its bioactive compounds induce apoptosis in various cancer cells through extrinsic and intrinsic apoptotic pathways. The green arrow points out the upregulation of signaling molecules, while the red arrow indicates the downregulation of molecular targets. DL: death ligand, DR: death receptor, TNF-*α*: tumor necrosis factor, FasL: Fas ligand, TRAIL: TNF-related apoptosis-inducing ligand, Bcl-2: B-cell lymphoma-2, Bad: Bcl-2-associated death promoter, Bax: Bcl-2-like protein, Apaf-1; apoptotic protease-activating factor 1.

The anticancer potential of Chinese propolis was studied on human breast cancer cells. Exposure to ethanolic Chinese propolis extract (EECP) significantly increased annexin A7 expression, ROS, NF-*κ*B, and p65 expressions and dramatically altered the potential of mitochondrial membrane [86]. Propolis, chrysin, and CAPE promote apoptosis in various cancer cells through both extrinsic and intrinsic apoptotic signaling (Figure 3). Neurofibromatosis (NF) is a genetic disease due to the defects in the NF1/NF2 gene. It shows an abnormal activation of an oncogenic kinase named PAK1, where the growth of both NF1 and NF2 tumors requires PAK1, and all PAK1 blockers, synthetic chemicals (the ring peptide FK228, UnPAK309 (PF3758309), a combination of two tyrosine-kinase inhibitors, PP1 and AG 879/GL-2003, and ivermectin), or natural products like curcumin, propolis, bitter melon, and berberine suppress the growth of these NF tumor cells *in vitro* and *in vivo* mice model [97]. Out of this, propolis extracts showed a boost in their therapeutic effect for NF patients without any side effects [98–100]. The ethanolic extract of Brazilian red propolis exhibits a strong cytotoxicity effect on breast cancer cells (MCF-7) and triggers apoptosis in these cells [101]. The combination treatment of propolis with radiation therapy prevents the leukocyte's DNA damage during the ionizing radiation in patients [102]. CAPE's cytotoxic and apoptotic effects on the RKO colorectal cancer cell line and the CCD 841-CoN normal colorectal cell line were recently examined. They discovered that CAPE caused apoptotic cell death in around 40% of the RKO cells. Furthermore, CAPE exposure increased p53 phosphorylation at Serine 15 and Serine 46 while decreasing survivin expression. The study indicated that CAPE promoted apoptosis through modulating p53 phosphorylation, resulting in the reduction of survivin expression, and that CAPE may be considered as an alternative treatment in cancer therapy [43].

The CAPE treatment showed strong cytotoxic activity on NPC cell lines. It also promotes apoptosis in NPC cells by downregulating the expression of antiapoptotic proteins Bcl-xL, elevating the expression of proapoptotic proteins Bax, and generating PARP cleavage [46]. Another study examined the anticancer properties of the CAPE analogue chemical 5A in nasopharyngeal carcinoma (NPC) cells. They discovered that chemical 5A effectively suppressed cell proliferation while having negligible cytotoxicity in normal cells. Moreover, compound 5A was discovered to promote cell cycle arrest and apoptosis in CNE2 cells by inhibiting the expression of EGFR downstream signaling molecules in NPC cells [44]. Egyptian propolis contains a high level of flavonoids, phenolics, and dihydroflavonoids in propolis extract, and it has anticancer effects in vitro studies and inhibits tumor growth in Ehrlich ascites carcinoma (EAC) in mice model. Propolis extract in combination with methotrexate stimulates the G0/G1 phase cell cycle arrest and induces apoptosis [8]. The latest study demonstrated that 25 to 100 *μ*g/ml of Chinese propolis-treated cells showed increased ROS generation and altered mitochondrial membrane potential, thereby it induced apoptosis in breast cancer cells [79].

Docetaxel with CAPE inhibited the proliferation and survival of docetaxel-resistant prostate cancer cells via inhibiting Bcl-2 and c-Myc and induced metabolic interference and apoptosis. Patients with docetaxel-resistant prostate cancer may benefit from a combination therapy [103].

Overall, all of these above research reported that propolis and its constituents induce both death ligand-mediated (Extrinsic) apoptotic pathway and mitochondrial-mediated (intrinsic) apoptotic pathway in various cancer cells by modulating the several apoptosis-related signaling molecules such as TRAIL, FasL, TNF-*α*/DR, p53, Bax, Bcl-2, Bcl-xL, caspases, and PARP (Figure 3).

### 3.3. Effect of Propolis and Its Bioactive Compounds on Angiogenesis and Cancer Metastasis

Angiogenesis or neovascularization is the development of fresh blood vessels from present ones, and this process is very important to regulate the growth and maintenance of metastatic tumors [104]. Angiogenesis is a very complicated process, which stimulates cell proliferation, migration, and invasion [105]. Therefore, tumor development is mainly dependent on the angiogenesis process. Lately, numerous studies have reported that the inhibitor for angiogenesis is evolved to treat cancer by targeting the multiple molecules and signaling pathways, which are regulating the angiogenesis process [106]. However, the commercially existing drugs are only effective against certain types of tumor due to the complexity of cancer, particularly cancer signaling and angiogenesis [107]. Angiogenesis is regulated via various signaling pathways, which are extremely complex and involve numerous angiogenic mediators. The important growth factors and mediators of angiogenesis include VEGF (vascular endothelial growth factor), PDGF (platelet-derived growth factor), FGF (fibroblast growth factor), EGF (epidermal growth factor), integrins, angiopoietins, endothelins, notch, ephrins, and cadherins [108]. Cancer metastasis is a process involving cell invasion, cell migration, cell adhesion, extracellular matrix (ECM), and degradation of basement membrane by proteinase enzymes. Matrix metalloproteinases (MMPs) such as MMP-2 and MMP-9 and intercellular adhesion molecule (ICAM) play important role in proteolytic degradation. Many studies have already showed the overexpression of MMPs in cancer tissues including breast, colon, lung, and pancreatic cancer. Also, uPA (urokinase-type plasminogen activator) is a serine protease, involved in proteolytic degradation during cancer invasion, migration, and metastasis [109]. Recently, Li et al. [79] reported that Chinese propolis (25 to 100 *μ*g/ml)-treated breast cancer cells showed decreased migration and invasion by targeting glycolysis enzymes and proinflammatory cytokines (TNF-*α*, IL-6, and NLRP3) in breast cancer cells.

Hwang et al. [110] have reported the impact of CAPE treatment on fibrosarcoma cells' (HT1080) invasion and metastasis. The results showed that CAPE treatment can suppress mRNA and protein expression of MMP-2 and MMP-9 in fibrosarcoma cells. Also, the mRNA expression of tissue inhibitor of matrix metalloproteinases (TIMPs) and MT-1 MMPs was decreased in CAPE-treated cells. Finally, it inhibits the colony formation potential, cell invasion, and migration. Cuban propolis (83 *μ*g/ml) suppresses cell migration and invasion by inhibiting MMP-9 activity, *β*-catenin, vimentin expression, and decreased E-cadherin expression in human colorectal cancer cells [111]. Red propolis produces antioxidant effects, also inhibits angiogenesis through the modulation of angiogenic factors and inflammation, and reduces the levels of VEGF and HIF-1*α*, and this shows the relationship between angiogenesis, oxidative stress, and tumor hypoxia [112, 113]. The association between the antiangiogenic and antioxidant effects of propolis was evaluated *in vitro* using endothelial cells; unsurprisingly, the most antiangiogenic compounds also had antioxidant properties [114]. Brazilian red propolis, a water-insoluble resinous mixture of the saliva of bees (Apis mellifera) and vegetable exudate, mainly from *Dalbergia ecastaphyllum* (L) Taub, has strong antioxidant activity and has been investigated and proposed as an inhibitor of angiogenesis [34, 115].

Another study demonstrated that red propolis and active compounds can alter the progression of carcinogenesis and induce cytotoxicity to lineages of tumor cells *in vitro* [116]. However, a study with tumor cell lineages revealed that different concentrations of red propolis are associated with different profiles of cytotoxicity [117]. Previous research has reported that the WSDP may have many biological effects including antitumor [118, 119], antioxidant [120], immunomodulatory [121], anti-infectious, and anti-inflammatory [122], and also, they identified the constituents of propolis (artepillin C, caffeic acid, and caffeic acid phenethyl ester) and studied their anticarcinogenic effects [123, 124]. Dornelas et al. [125] have extracted WSDP from soluble propolis using 8% L-lysine. They have reported that green propolis WSDP may inhibit angiogenesis in N-butyl-(-4-hydroxybutyl) nitrosamine-stimulated bladder cancer model in rats, while L-lysine treatment induces angiogenesis if started simultaneously with N-butyl-(-4-hydroxybutyl) nitrosamine.

Jung and co-workers conducted an extensive study using caffeic acid, in which caffeic acid inhibited the tube formation in HUVEC (human umbilical vein endothelial) cells in 3D culture, and also, this treatment inhibited the angiogenesis of human kidney tumor implanted in nude mice. Furthermore, they found the reduction of VEGF and slowdown of tumor growth by inhibiting the STAT phosphorylation and decreasing the HIF-1-mediated expression of VEGF [126]. In another study, Brazilian green propolis and its active compound caffeic acid derivatives inhibited VEGF-induced cell migration and tube formation in HUVECs [127]. Caffeic acid inhibits human retinal tumor angiogenesis by blocking VEGF expression through STAT-3 [112]. All of the above studies suggest that the caffeoyl group's compounds in propolis may be the reason for its anticancer activities. Chinese red propolis and CAPE displayed a solid inhibitory effect in VEGF-mediated angiogenesis. The study suggested that Chinese red propolis and CAPE may act as a strong antiangiogenic therapeutic agents for human diseases [112]. Liao et al. [128] described that CAPE may be a potential inhibitor for angiogenesis and also suppress neovascularization. The VEGF expression significantly decreased in CAPE-treated colon cancer cells (CT26) and also inhibited the synthesis of prostaglandin E2. All of these studies have reported the anticancer activities of propolis and its well-known active components including caffeic acid, CAPE, chrysin, and artepillin C. Most *in vitro* and *in vivo* studies demonstrated that propolis targets various growth factor signaling, thereby it inhibits cell proliferation, cell cycle progression, cancer cell migration, and invasion and induces apoptosis in cancer cells. In a recent study, we demonstrate that a low-dose combination of Wi-A and CAPE causes reversal of EMT by the upregulation of E-cadherin and Claudin1, yielding inhibition of Wnt/*β*-catenin, vimentin, MMPs, VEGF, and VEGFR signaling pathways in cervical and breast cancer cells [129].

According to another recent study, the expression of GSK-3*β* suppressed Snail and the disruption of HDAC6-mediated vimentin protein stability inhibits the cell migration and invasion of TNBC by propolin G. Propolin G, which targets EMT, might be a contender for TNBC treatment [130]. Furthermore, Niyomtham et al. discovered that the ethyl acetate extract of propolis (EAEP) was cytotoxic and caused apoptosis in HNSCC cell lines. In addition, EAEP inhibited HNSCC cell invasion by lowering MMP-2 and MMP-9 activities. HPLC-ESI-TOF-MS was used to identify two flavonoids in EAEP: galangin and apigenin. The findings imply that EAEP causes apoptosis and has anti-invasion properties in HNSCC cell lines via reducing MMP-2 and MMP-9 activities. Galangin and apigenin may be responsible for these inhibitory actions [131]. In another study, CAPE hinders the migratory and invasive ability of NPC cells by reversing the epithelial-mesenchymal transition (EMT) pathway by modulating the EMT marker protein expression. The CAPE exposure upregulates the E-cadherin expression and downregulates the mesenchymal marker (vimentin, *β*-catenin, and MMP) in NPC cells [46]. Another recent study demonstrated that Chinese propolis (12.5 *μ*g/ml) inhibited Panc-1 cell migration by modulating the epithelial-mesenchymal transition. Interestingly, after CP treatment, the Hippo pathway was stimulated in Panc-1 cells, providing as a mechanism for CP's antipancreatic cancer activity [132]. Overall, the studies have found that propolis and its constituents inhibit cell migration, epithelial-to-mesenchymal transition, and tumor metastasis by regulating several signaling molecules such as growth factors (EGF, VEGF, PDGF, HIF-1, and FGF), EMT markers (vimentin, E-cadherin, *β*-catenin, and Snail), and metastatic-related proteins (MMPs, uPA, and interleukin).

## 4. Summary and Conclusions

Overall, the present review highlights the anticancer activities and molecular targets of propolis in different cancer cells. The chemical composition of propolis differs greatly among species of bees and depends on geographical and climatic factors, plant resources, and collecting seasons, and it requires a different experimental approach to evaluate their biological potential. We summarized the inhibitory effect of propolis on cancer cell proliferation and metastasis-related signaling pathways, and also propolis induces apoptosis by targeting both extrinsic and mitochondrial-mediated apoptotic signaling pathways. Propolis has active ingredients such as CAPE, chrysin, and artepillin C. The antioxidant, antiproliferative, antiangiogenic, and antimetastatic potential of propolis were attributed to the presence of the above active compounds. Propolis' anticancer mechanisms are diverse, since it operates on a variety of cancer metabolic targets. Preventing metastatic spread, blocking nuclear localization of NF-*κ*B, gene expression regulation, inactivation of MMP, and activation of tumor suppressors, and overcoming TRAIL resistance of cancer cells have all been identified as critical pathways. Clinical trials require standardized quality control and perfect design, and metabolomics is widely regarded to help achieve these goals. With the right research, novel, low-cost cancer therapies can be produced from propolis. The bioactive compounds of propolis, and the target molecules involved, must be investigated further in order to create novel cancer therapeutics and overcome the problem of chemotherapy resistance. Therefore, propolis, a natural compound, might be suggested for the combination therapy to reduce the adverse effect of chemotherapy. However, further clinical studies are needed to validate the ratio of propolis compounds in human subjects and also allergenic reactions to propolis, and its dosage merits require further research.

## Data Availability

No data were used to support this study.
